# Idiopathic pulmonary haemosiderosis with mineralizing pulmonary elastosis: A case report

**DOI:** 10.1186/1752-1947-2-65

**Published:** 2008-02-27

**Authors:** Amanjit Bal, Ashish Bhalla, Kusum Joshi

**Affiliations:** 1 Department of Histopathology, PGIMER, Sector-12, Chandigarh-160012, India; 2 Department of Internal Medicine, PGIMER, Sector-12, Chandigarh-160012, India

## Abstract

**Introduction:**

Idiopathic pulmonary haemosiderosis characterized by repeated episodes of intra-alveolar haemorrhage is rare in adults and has a relatively benign course compared to cases seen in children.

**Case Presentation:**

The case presented here is of an adult man with idiopathic pulmonary haemosiderosis with mineralizing pulmonary elastosis.

**Conclusion:**

Pathologists are generally not familiar with this histologic reaction pattern associated with iron encrustation of pulmonary elastic tissue.

## Introduction

Diffuse pulmonary haemosiderosis is characterized by repeated episodes of intra-alveolar haemorrhage leading to abnormal accumulation of iron as haemosiderin in alveolar macrophages with subsequent pulmonary fibrosis and severe anaemia [1,2]. Pulmonary haemosiderosis (PH) occurs either primarily as a disease of the lungs or secondary to systemic diseases. Idiopathic PH, first described by Ceelen in 1931, is characterized by a clinical triad of haemoptysis, anaemia and pulmonary infiltrates. Eighty percent of cases of PH occur in children [2]. We report a case of an adult man with idiopathic PH with mineralizing pulmonary elastosis.

## Case presentation

A 32-year-old, non-smoking, male farmer presented with a history of fever, intermittent episodes of mild haemoptysis and cough with expectoration for the previous six months. There was associated loss of appetite and loss of weight. He started experiencing respiratory distress 8 days prior to hospital admission. Two years earlier he was diagnosed with pulmonary tuberculosis based on X-ray findings whereupon he was commenced on anti-tubercular treatment, however the level compliance is not known. On physical examination his respiratory rate and jugular venous pressure were elevated, and he had clubbing. On chest auscultation there were bilateral coarse crepitations. Pulmonary function tests were consistent with restrictive ventilatory defect. Laboratory investigations revealed iron deficiency anaemia and there were negative results for antinuclear antibodies (ANA) and antineutrophilic cytoplasmic antibodies (ANCA). On ultrasound examination of the abdomen there was evidence of hepatosplenomegaly. The clinical impression was of disseminated tuberculosis. The patient was placed on ambu ventilation and was managed with anti-tubercular treatment, antibiotics and intravenous fluids. The patient succumbed to his illness 10 days after admission to hospital as a result of type II respiratory failure.

### Autopsy findings

**Figure 1:**
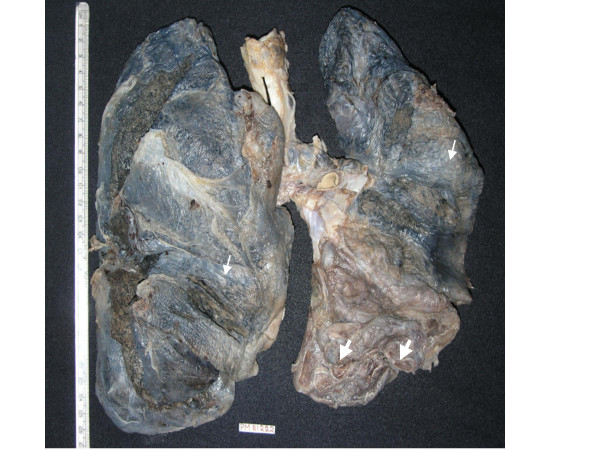
Gross photograph showing dull, opaque, and thickened pleura, vague nodular lesions (Thin arrows) and a collapsed left lower lobe with honeycomb changes (Thick arrows).

**Figure 2:**
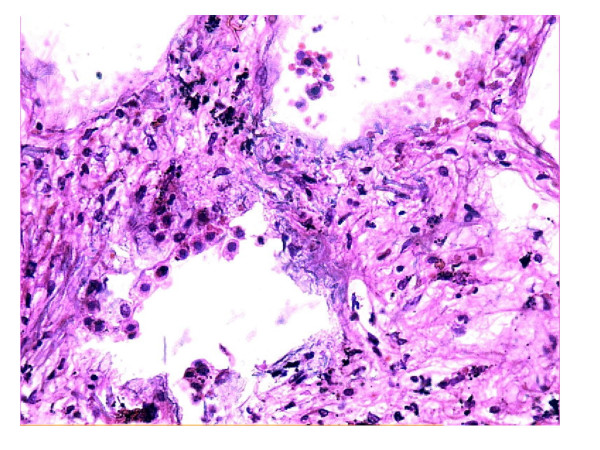
Photomicrograph showing vascular, interstitial and alveolar elastic fibres encrusted with gray-blue to golden refractile deposits (H&E, ×200).

**Figure 3:**
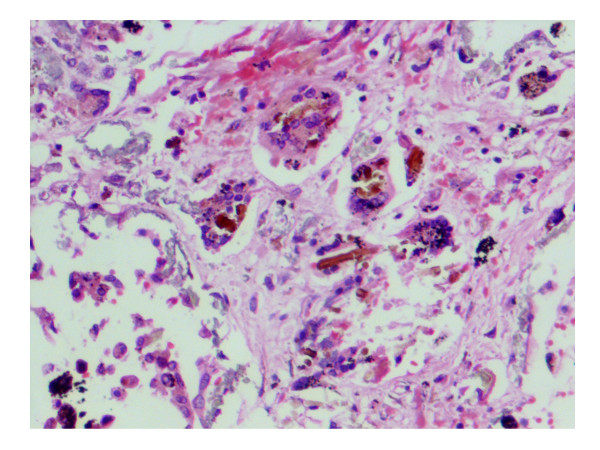
Photomicrograph showing fractured deposits with vague rod shaped structures resembling asbestos bodies and eliciting a foreign body giant cell reaction (H&E, ×200).

**Figure 4:**
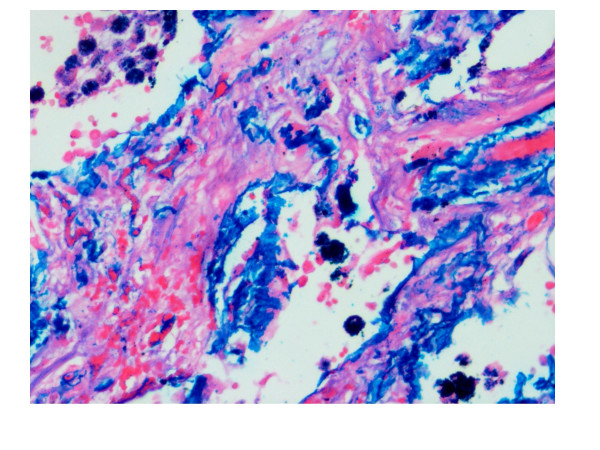
Photomicrograph showing alveolar spaces containing haemosiderin-laden macrophages and the mineral deposits positive for Prussian blue reaction (Perls' stain, ×200).

**Figure 5:**
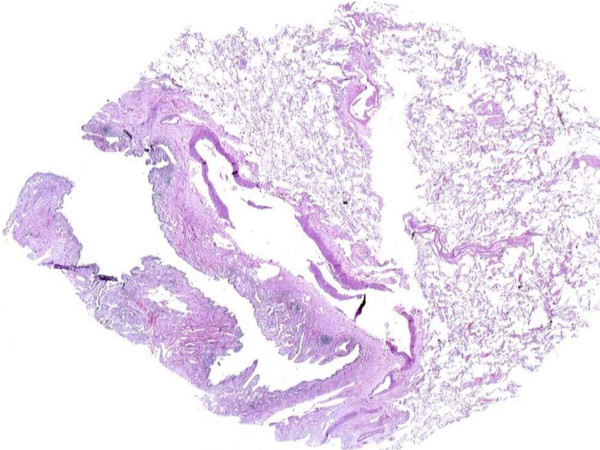
Photomicrograph showing a scanner view highlighting cystically dilated bronchioles (H&E, ×20).

A complete autopsy was performed. On opening the thoracic cavity there was obliteration of the left pleural cavity while the pericardial cavity yielded around 250 ml of blood tinged fluid. Both the lungs together weighed 2000 grams, were heavily pigmented, and had a subcrepitent feel. The pleura were dull, opaque, thickened and adherent. The left lower lobe was collapsed with honeycomb change and on cut surface the left lower lobe showed cysts ranging in size from 0.5 cm – 1 cm diameter along with prominent bronchioles (Fig. 1). The rest of the lung parenchyma showed haemorraghic consolidation. The right lower lobe showed mild honeycombing and haemorraghic consolidation of the rest of the parenchyma. In addition both lungs on palpation revealed grayish white nodules of 0.2 cm in diameter. The pulmonary vessels were dilated and could be traced to the sub-pleural surface. On microscopic examination there was thickening of alveolar septa with interstitial fibrosis. Vascular and alveolar elastic fibres were encrusted with grey-blue to golden refractile mineral deposits (Fig. 2). Some of the deposits were fractured into vague rod shaped structures resembling asbestos bodies and elicited foreign body giant cell reaction (Fig. 3). Alveolar spaces contained haemosiderin-laden macrophages. These mineral deposits were positive for Prussian blue reaction (Fig. 4) and von Kossa stain. Additionally lung parenchyma showed marked capillary congestion, ectasia and proliferation. Foci of fresh intra-alveolar haemorrhages were also present. There was no evidence of granulomas, capillaritis, vasculitis or other organic lung disease. Superadded bronchopneumonia was seen in both the lungs. The cystic spaces in the left lung showed changes of bronchiectasis (Fig. 5). Electron microscopic examination of lung tissue showed focal thickening and scattered foci of thinning of the alveolar capillary basement membrane however there were no electron dense deposits. The heart weighed 340 gm and was enlarged and globular with dullness of the pericardium. There was marked dilatation of the right atrium, left atrium, coronary sinus, right ventricular cavity and pulmonary trunk. There was right ventricular hypertrophy with wall thickness of 1 cm. The liver and spleen showed changes of chronic venous congestion. The brain weighed 1000 gm and showed marked congestion of the meninges and both gray and white matter. The right putamen and thalamus showed slight discoloration. On microscopic examination there were hypoxic changes and a focal area of haemorrhage with haemosiderin-laden macrophages in the basal ganglia. The kidneys showed changes consistent with acute tubular necrosis. The gastrointestinal tract was grossly and microscopically within normal limits. The bone marrow revealed hyperplastic erythropoiesis and low iron stores.

Final autopsy diagnosis was of idiopathic pulmonary haemosiderosis with mineralizing pulmonary elastosis.

## Discussion

Pulmonary haemosiderosis is characterized by repeated episodes of intra-alveolar haemorrhage that lead to abnormal accumulation of iron as haemosiderin in alveolar macrophages. PH can occur as a primary disease of the lungs, or secondary to systemic diseases which can be divided into 3 broad groups[2]; Group I: Pulmonary haemorrhage and immune complex diseases such as Wegener's granulomatosis, systemic lupus erythematosus, rheumatoid arthritis; Group II: Pulmonary haemorrhage and Goodpasture's syndrome; Group III: Includes coagulopathies, platelet defects, pulmonary infections, pulmonary neoplasms, pulmonary veno-occlusive disease, pulmonary capillary haemangiomatosis, mitral stenosis, exposure to toxins such as cocaine, pesticides or insecticide, and idiopathic cases.

Idiopathic PH, first described by Ceelen in 1931, is a rare condition. About 80% of cases occur in children. There is slight male predominance in adult onset idiopathic PH [3]. In the acute phase, patients present clinically with a triad of haemoptysis, anaemia and pulmonary infiltrates. However, in the chronic phase the predominant findings are pallor, failure to thrive, hepatosplenomegaly, crackles and clubbing [4]. The man described in this report presented with features consistent with the chronic phase. As the name suggests its aetiopathogenesis is unknown but various aetiological hypotheses have been proposed which include autoimmune, allergic, metabolic and environmental causes [5,6]. In this case the patient was a farmer by occupation, so exposure to insecticides could be a possible aetiology.

Iron encrustation of pulmonary elastic tissue is associated with recurrent pulmonary haemorrhages, most notably in idiopathic PH and pulmonary veno-occlusive disease. Lendrum [7] in 1950 described iron encrustation of pulmonary elastica in patients with cardiac disease, however, pathologists are generally not familiar with this histologic reaction pattern. Although the term iron encrustation is commonly applied, histochemical examination confirms the simultaneous presence of calcium phosphate as well. Thus the term "mineralizing pulmonary elastosis" is preferred over "iron encrustation of elastica" or "endogeneous pneumoconiosis" [8]. The pathogenesis of mineralizing elastosis is not known but it is speculated that the primary event is alveolar haemorrhage. Breakdown of erythrocytes results in the formation of ferric and ferrous ions which are present close to pulmonary connective tissue. The native affinity for minerals and degenerative changes in elastin predisposes to mineralization. Mineralization further intensifies the vascular damage and leak. These ferruginous elastin fibres resemble asbestos bodies and are a source of diagnostic error. However, unlike asbestos bodies, they lack symmetry or a beaded appearance.

## Conclusion

Idiopathic PH is rare in adults and one probable aetiology is direct exposure to insecticides. Pathologists are generally unfamiliar with the infrequently seen histologic reaction pattern of "mineralizing pulmonary elastosis" which is seen in cases of recurrent pulmonary haemorrhage and is a source of diagnostic confusion. Elastic fibre encrustation contributes to lung restriction and accelerated interstitial injury.

## Competing interests

The author(s) declare that they have no competing interests.

## Authors' contributions

ABa participated in the histopathological diagnosis, writing of the manuscript and photomicrography. ABh provided the clinical details of the patient. KJ participated in the histopathological diagnosis, and editing of the manuscript. All authors read and approved the final manuscript.

## Consent

Written informed consent was obtained from the patient's next of kin for publication of this case report and any accompanying images which are based upon an autopsy. A copy of the written consent is available for review from the Editor-in-Chief of this journal
